# Predictive value of circulating SNHG1/miR-194-5p and carotid ultrasound for the onset of cerebral infarction in patients with transient cerebral ischemia

**DOI:** 10.3389/fnmol.2025.1689127

**Published:** 2025-11-19

**Authors:** Wei Wang, Mingjia Lv, Xin Zhang

**Affiliations:** Department of Ultrasound Examination, Shengli Oilfield Central Hospital, Dongying, Shandong, China

**Keywords:** carotid ultrasound, transient cerebral ischemia, cerebral infarction, miR-194-5p, gene

## Abstract

**Background:**

Transient cerebral ischemia is a strong warning sign of cerebral infarction (CI). Early objective risk assessment in patients with transient cerebral ischemia can effectively help prevent the occurrence of CI.

**Objective:**

The study aimed to explore the predictive value of SNHG1/miR-194-5p in combination with carotid ultrasound for predicting the occurrence of CI in patients with transient cerebral ischemia.

**Patients and methods:**

This study was a prospective observational study. A total of 189 patients with transient cerebral ischemia were included and divided into the CI group (n = 67) and the non-CI group (n = 122) based on whether CI occurred within 90 days. The clinical data and laboratory indexes of the two groups were compared. RT-qPCR was employed to examine the levels of SNHG1/miR-194-5p. Logistic regression analysis and receiver operating characteristic (ROC) curve analysis were performed based on serum SNHG1/ miR-194-5p levels and the degree of carotid artery stenosis. In addition, bioinformatics analysis was carried out to identify the target genes of miR-194-5p.

**Results:**

The results showed that, compared to the non-CI group, the expression of SNHG1 in the serum of the CI group was upregulated, while the expression of miR-194-5p was downregulated. Logistic regression analysis showed that the expression of miR-194-5p (OR = 0.067, *p* < 0.001) and SNHG1 (OR = 25.984, *p* < 0.001) and the degree of carotid artery stenosis (OR = 1.152, *p* = 0.001) were significantly correlated with CI. The combined detection of these three indicators yielded an AUC value of 0.953 for predicting CI. Its sensitivity was 89.55% and specificity was 86.89%, indicating higher diagnostic efficiency than any single indicator. Furthermore, bioinformatics analysis revealed that the target gene of miR-194-5p was enriched in various disease pathways, especially those related to neurodegeneration, providing a new direction for exploring the mechanism of CI.

**Conclusion:**

Serum SNHG1/miR-194-5p levels combined with carotid ultrasound show high predictive accuracy for the short-term occurrence of CI in patients with transient cerebral ischemia.

## Introduction

Cerebral infarction (CI) is a common cerebrovascular disease that seriously threatens human health and quality of life (Zhao et al., 2022; Lakshmipriya and Gopinath, 2025). Transient cerebral ischemia is closely related to CI (Lidong et al., 2021; Hasegawa et al., 2021). Transient cerebral ischemia refers to neurological dysfunction caused by a transient insufficiency of blood supply to the brain (Warach and Schellinger, 2024; Steliga et al., 2023). The symptoms usually resolve completely after an attack and are often overlooked by patients (Rutkai et al., 2022). Currently, there are numerous and diverse approaches available for predicting the risk of CI in clinical practice. Analyzing key clinical risk factors, such as hypertension, diabetes, and hyperlipidemia, is an essential component of these approaches (Yuan et al., 2018). Although these factors can provide some reference basis, they still cannot accurately predict the risk of cerebral infarction. Imaging examinations can visually display brain lesions, but their predictive value for early CI is limited. Therefore, identifying a more accurate and effective predictive indicator has become an urgent challenge in clinical practice.

In recent years, the long non-coding RNA (lncRNA) SNHG1, a novel biomarker, has been shown to be involved in the development of various diseases (Thin et al., 2019; Xiao et al., 2022). Studies have found that SNHG1 is abnormally expressed in various tumor tissues and cells and is closely related to tumor proliferation, invasion, and metastasis (Yang et al., 2023; Fonseca et al., 2024). In the field of cardiovascular diseases, SNHG1 has also been reported to be related to the occurrence and development of diseases, such as atherosclerosis and myocardial infarction (Liu et al., 2024; Lu et al., 2020). However, the expression level of SNHG1 in patients with transient cerebral ischemia and its relationship with the occurrence of CI have not yet been reported. MicroRNAs (miRNAs) are endogenous non-coding RNAs that regulate gene expression at the post-transcriptional level through complementary pairing and binding with target genes (Paraskevopoulou and Hatzigeorgiou, 2016). miR-194-5p is an important miRNA, which has been confirmed to be involved in various physiological and pathological processes (Clement et al., 2023; Niu et al., 2021). Studies have shown that the expression level of miR-194-5p in the serum of patients with CI is significantly decreased, and it is closely related to the severity and prognosis of CI (Zou et al., 2019). However, the changes in miR-194-5p expression in patients with transient cerebral ischemia and its relationship with the occurrence of CI remain unclear.

Carotid ultrasound examination is a non-invasive, convenient, and economic examination method that has been widely used in clinical practice (Scoutt and Gunabushanam, 2019; Sultan et al., 2023). Studies have shown that carotid ultrasound examination can provide an important basis for the diagnosis, treatment, and prognosis evaluation of CI (Wu et al., 2022; Ni et al., 2020). However, relying solely on carotid ultrasound examination to predict the occurrence of CI still has certain limitations, and its accuracy needs to be further improved.

**Figure 1:**
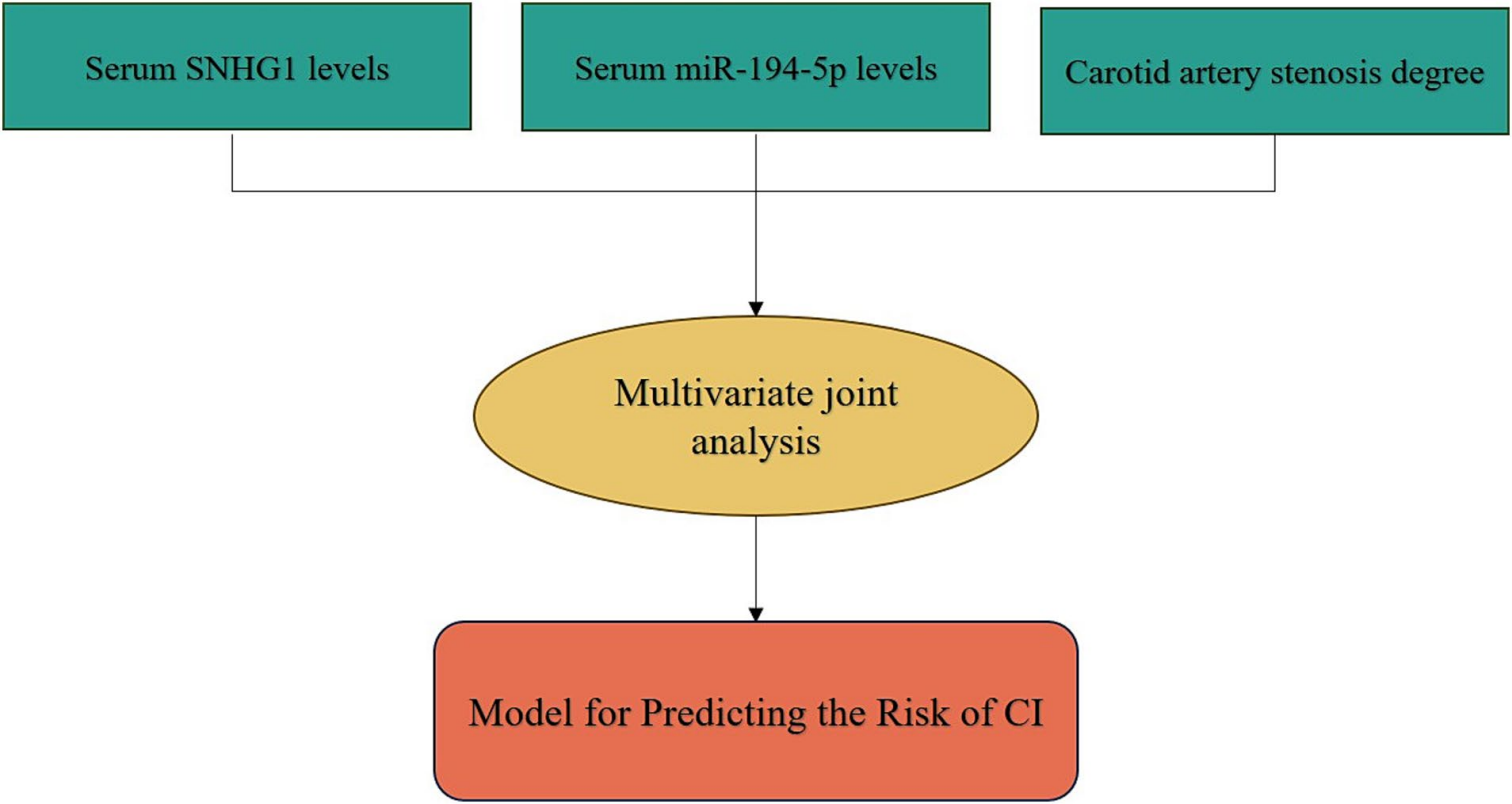
The model framework for predicting CI in patients with transient ischemic attack by combining serum SNHG1/miR-194-5p and carotid ultrasound.

This study aimed to investigate the predictive value of circulating SNHG1/miR-194-5p combined with carotid ultrasound for the occurrence of CI in patients with transient cerebral ischemia. It sought to provide new strategies for the early clinical identification of high-risk CI patients and to offer a theoretical basis for personalized prevention and treatment. We detected the expression levels of SNHG1 and miR-194-5p in the serum of patients with transient cerebral ischemia and constructed a combined prediction model by integrating carotid ultrasound findings. The core framework is shown in Figure 1. This approach aimed to provide more precise and effective strategies for the early prevention and treatment of CI.

## Materials and methods

### Study population

A total of 189 patients with transient cerebral ischemia who were diagnosed and treated at Shengli Oilfield Central Hospital from March 2022 to August 2024 were included in the study. They were divided into two groups based on the occurrence of secondary CI within 90 days. Patients with secondary CI were classified as the CI group (*n* = 67), and those without secondary CI were classified as the non-CI group (*n* = 122).

The inclusion criteria were as follows: (1) meeting the diagnostic criteria for transient cerebral ischemia and CI; (2) presentation within 24 h of symptom onset; (3) normal function of major organs and systems, as confirmed by clinical examinations; and (4) complete and traceable data for all examinations in this study. The exclusion criteria included the following: (1) severe functional disorders of major organs, such as heart failure, liver failure, and renal failure, (2) presence of malignant tumors, being in an advanced stage of the disease, or undergoing radiotherapy and chemotherapy; and (3) hematologic disorders, such as leukemia and thrombocytopenic purpura, that may affect the detection of blood indicators. Venous blood was collected from the participants at the time of admission, and serum samples were isolated by centrifugation and stored at −80 °C for subsequent analyses. All patients and their families signed the informed consent form. This study was approved by the Medical Ethics Committee of Shengli Oilfield Central Hospital.

### Clinical data collection

The baseline data of the participants, including age, gender, BMI, history of smoking and drinking, and diabetes, were recorded for analysis. Laboratory parameters were also obtained and analyzed, including total cholesterol (TC), triglyceride (TG), low-density lipoprotein cholesterol (LDL-C), and high-density lipoprotein cholesterol (HDL-C).

### Detection of serum SNHG1/miR-194-5p expression levels

Total RNA was isolated and purified from the serum using the RNeasy Mini Kit (Qiagen, Valencia, CA), and the RNA was reverse transcribed into cDNA using a reverse transcription kit (Takara, Tokyo, Japan). qPCR was conducted using the SYBR Premix Ex TaqTM II Kit (Takara, Tokyo, Japan) and an American ABI7500 Fluorescent Quantitative PCR instrument. GAPDH was used as the internal reference gene for SNHG1, and U6 was used as the internal reference gene for miR-194-5p. Normalization correction was carried out. All qPCR reactions were subjected to three rounds of repeated experiments, and the results were presented as the mean ± standard deviation (SD). The mean values after the three replicates were calculated using the 2^-ΔΔCt^ method. The primer sequences used in the reactions were synthesized by GenePharma (Shanghai, China) and are listed in Table 1.

**Table 1:** Primer sequences for RT-qRCP.

Genes	Primer sequences (5′-3′)
SNHG1 (forward)	TAACCTGCTTGGCTCAAAGGG
SNHG1 (reverse)	CAGCCTGGAGTGAACACAGA
miR-194-5p (forward)	GCCGTGTAACAGCAACTCCA
miR-194-5p (reverse)	GTGCAGGGTCCGAGGT
GAPHD (forward)	AGGTCGGTGTGAACGGATTTG
GAPHD (reverse)	TGTAGACCATGTAGTTGAGGTCA
U6 (forward)	GCTTCGGCAGCACATATACTAAAAT
U6 (reverse)	CGCTTCACGAATTTGCGTGTCAT

miR-194-5p, microRNA-194-5p; SNHG1, long noncoding RNA SNHG1; GAPHD, glyceraldehyde-3-phosphate dehydrogenase.

### Carotid ultrasound examination

A blinded design was adopted for carotid ultrasound examinations, and all operators and result interpreters were unaware of the participants’ biomarker test results. The Resona R9 color Doppler ultrasound diagnostic machine (Mindray, Shenzhen, China) and the L14-3WU linear array probe were used. The bilateral common carotid arteries and the internal and external carotid arteries of the patients were examined. The degree of stenosis was measured at the narrowest part of the lumen and expressed as a percentage. According to the relevant diagnostic criteria of the symptomatic carotid endarterectomy method in North America (Ravin et al., 2015), carotid artery stenosis degree was classified into four grades: A stenosis rate > 99% was defined as complete occlusion, a stenosis rate ranging from 70 to 99% was classified as severe stenosis, a stenosis rate ranging from 50 to 69% was considered moderate stenosis, and a stenosis rate < 50% was regarded as mild stenosis.

### Dual-luciferase reporter gene assay

Using starBase^1^, the potential binding sites between SNHG1 and miR-194-5p were predicted. To verify their interaction, based on the predicted complementary binding sequences of miR-194-5p and SNHG1, dual-luciferase reporter recombinant plasmids, SNHG1-WT (containing wild-type binding sites) and SNHG1-MUT (containing mutant binding sites), were synthesized. The recombinant plasmids were co-transfected into SH-SY5Y cells using Lipofectamine 3,000, along with the following controls: mimic NC, miR-194-5p mimic, inhibitor NC, and miR-194-5p inhibitor. After 48 h, relative luciferase activity was evaluated using the dual-luciferase reporter assay system (Promega), and the results were normalized to Renilla luciferase activity.

### Bioinformatics analysis

We used the miRWalk, miRDB, and TargetScan databases to analyze and predict the target genes of miR-194a-5p. The predicted target genes were visualized as Venn diagrams using Venny2.1.0. Overlapping target genes were then uploaded to the STRING online database to establish a protein–protein interaction (PPI) network. Subsequently, the top 10 hub genes were selected using the cytoHubba plugin in Cytoscape. Gene Ontology (GO) and the Kyoto Encyclopedia of Genes and Genomes (KEGG) were used to conduct gene description and pathway enrichment analysis on the overlapping target genes. In addition, bioinformatics tools^2^ were used, based on the gene sequences of the overlapping target genes, for data visualization and integration.

### Statistical analysis

The data were presented as mean ± SD. Student’s *t*-test and one-way ANOVA were used to assess between-group differences. A chi-squared test was applied to analyze the association between clinical features. The correlations between SNHG1, miR-194-5p levels, and carotid artery stenosis degree were analyzed using Pearson correlation coefficients. Multivariate logistic regression was performed to identify the factors influencing secondary CI in patients with transient cerebral ischemia. The receiver operating characteristic (ROC) curve was used to evaluate the predictive value of carotid artery stenosis degree, SNHG1, and miR-194-5p levels, as well as their combination, for secondary CI in patients with transient cerebral ischemia. A *p*-value of <0.05 was considered statistically significant.

## Results

### Baseline information of the study participants

The clinical characteristics of the CI group and the non-CI group are summarized in Table 2. There were no differences between the two groups in terms of gender, age, BMI, blood cholesterol, blood triglycerides, blood low-density cholesterol, smoking history, hypertension, and diabetes (*p* > 0.05).

**Table 2:** Baseline data and laboratory indicators of the participants.

Variables		Non - cerebral infarction group (*n* = 122)	Cerebral infarction group (*n* = 67)	*p* value
Age (year)		65.62 ± 5.61	64.90 ± 6.85	1.382
Gender	Male	58	37	0.312
	Female	64	30	
BMI (kg/m^2^)		23.15 ± 4.07	24.99 ± 5.23	9.101
Smoking history		82	36	0.067
Hypertension		41	28	0.264
Diabetes		98	49	0.255
TG (mmol/L)		3.11 ± 0.56	3.09 ± 0.58	0.297
TC (mmol/L)		4.14 ± 0.57	4.35 ± 0.62	1.766
LDL-C (mmol/L)		3.98 ± 0.16	4.02 ± 0.13	2.865
HDL-C (mmol/L)		0.97 ± 0.24	0.93 ± 0.20	2.477

Continuous data with normal and non-normal distribution are presented as mean ± standard deviation; TG, triglyceride; TC, total cholesterol; LDL-C, low density lipoprotein-cholesterol; HDL-C, high density lipoprotein-cholesterol.

### Serum SNHG1/miR-194-5p levels

**Figure 2:**
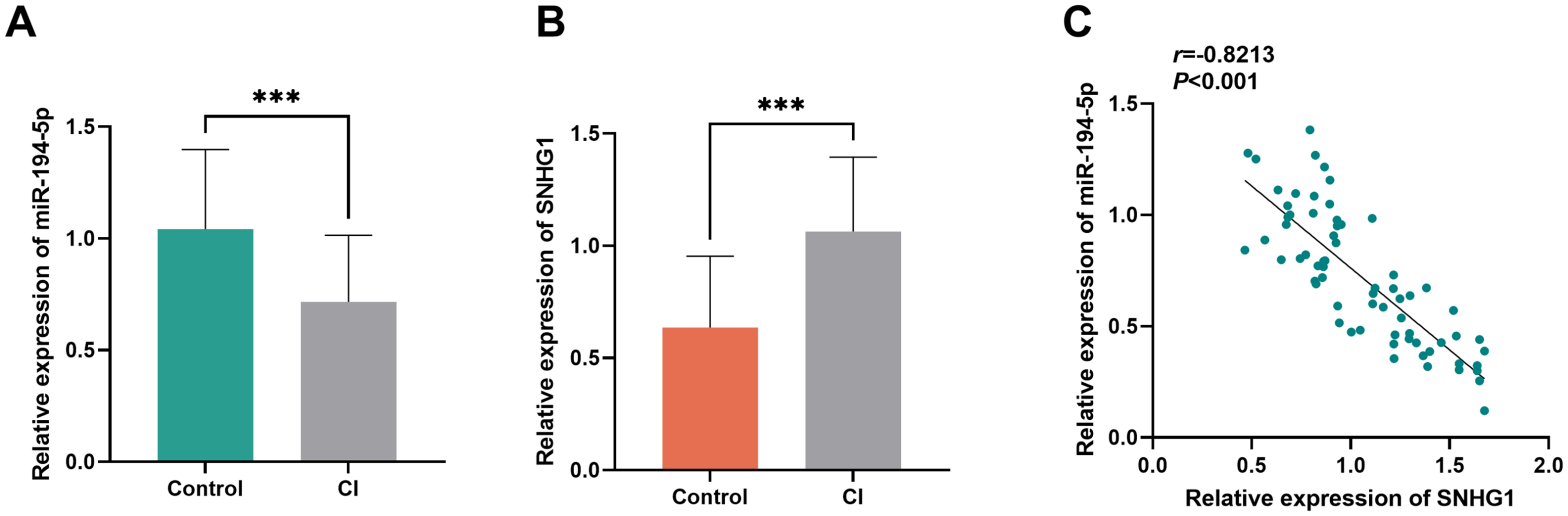
Expression and correlation analysis of SNHG1/miR-194-5p in the serum of the patients with CI. **(A)** Serum miR-194-5p was decreased in the patients with CI. **(B)** Serum SNHG1 was increased in the patients with CP. **(C)** The expression of miR-194-5p and SNHG1 was negatively correlated in the patients with CI. ***p < 0 0.001.

RT-qPCR analysis showed that, compared to the non-CI group, the expression of SNHG1 in the serum of the patients in the CI group was significantly upregulated (Figure 2A, *p* < 0.001). However, compared to the non-CI group, the expression of miR-194-5p in the serum of the patients with CI was significantly downregulated (Figure 2B, *p* < 0.001). According to the correlation analysis, miR-194-5p expression was negatively correlated with SNHG1 expression (*r* = −0.8213, *p* < 0.001, Figure 2C).

### Risk factors for CI in the patients with transient cerebral ischemia

We included age, gender, BMI, smoking status, drinking status, diabetes, TC, TG, LDL-C, HDL-C, the serum levels of SNHG1 and miR-194-5p, and carotid artery stenosis degree in the univariate logistic regression analysis. The results showed that TC, serum SNHG1 levels, miR-194-5p levels, and carotid artery stenosis degree showed statistically significant differences (*p* < 0.05) (Table 3). Factors with a *p*-value of <0.05 in the univariate regression analysis were included in the multivariate regression analysis. The multivariate logistic regression analysis showed (Table 4) that the degree of carotid artery stenosis (OR = 1.152, 95% CI = 1.098–1.210, *p* < 0.001) and the expression levels of SNHG1 (OR = 25.984, 95% CI = 5.513–122.474, *p* < 0.001) were independent risk factors for CI, while the expression level of miR-194-5p (OR = 0.067, 95% CI = 0.013–0.343, *p* = 0.001) was a protective factor.

**Table 3:** Univariate analysis of general information and detection indicators of patients with transient cerebral ischemia.

Factors	B	Wald *χ*2	OR (95% CI)	*p* value
Age	0.013	0.044	1.013 (0.930–1.104)	0.765
Gender	0.290	0.754	1.336 (0.305–5.860)	0.701
BMI	0.089	0.066	1.093 (0.959–1.244)	0.182
Smoking	−0.267	0.775	0.766 (0.168–3.498)	0.731
Hypertension	−0.105	0.764	0.900 (0.201–4.021)	0.890
Diabetes	−0.297	0.855	0.743 (0.139–3.973)	0.729
TG	−0.438	0.517	0.645 (0.234–1.777)	0.397
TC	1.555	0.728	4.737 (1.138–19.714)	0.033
LDL	−1.849	2.271	0.157 (0.002–13.487)	0.415
HDL	−0.949	1.393	0.387 (0.025–5.940)	0.496
Carotid artery stenosis degree	0.171	0.034	1.186 (1.109–1.268)	0.000
SNHG1	3.501	0.878	33.163 (5.933–185.384)	0.000
miR-194-5p	−3.181	1.055	0.042 (0.005–0.328)	0.003

**Table 4:** Logistic regression of influencing factors of cerebral infarction in patients with transient cerebral ischemia.

Factors	B	Wald *χ*2	OR (95% CI)	*p* value
Carotid artery stenosis degree (X1)	0.142	0.025	1.152 (1.098–1.210)	0.000
SNHG1 (X2)	3.257	0.791	25.984 (5.513–122.474)	0.000
miR-194-5p (X3)	−2.709	0.837	0.067 (0.013–0.343)	0.001

### Construction of the logistic prediction model

Multivariate logistic regression analysis identified three predictive factors: the expression level of serum SNHG1, the expression level of serum miR-194-5p, and the degree of carotid artery stenosis. Based on these three factors, a logistic prediction model was constructed as follows: *p* = 1/[1 + e^-(−6.743 + 0.142×1 + 3.257×2-2.709×3)^], where p represents the probability of secondary CI; X1 denotes carotid artery stenosis degree; X2 denotes the serum SNHG1 expression level; and X3 denotes the serum miR-194-5p expression level. A p-value closer to 1 indicated a higher likelihood of CI, whereas a p-value closer to 0 indicated a lower likelihood of CI.

### Predictive accuracy of risk factors for CI occurrence

**Figure 3:**
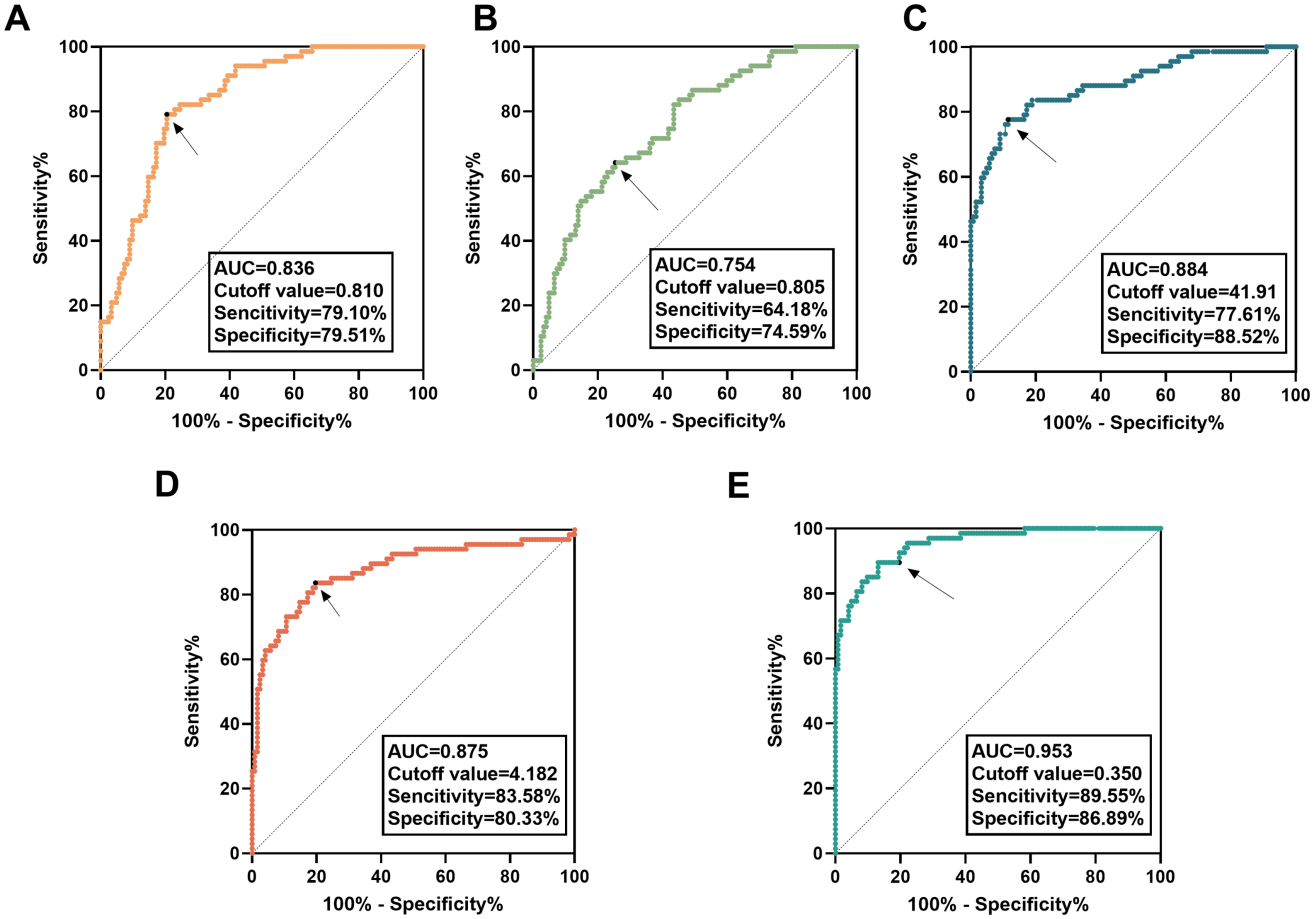
ROC curve analysis of SNHG1/miR-194-5p and carotid artery stenosis degree. **(A)** The ROC curve of miR-194-5p. **(B)** The ROC curve of SNHG1. **(C)** The ROC curve of carotid artery stenosis degree. **(D)** The ROC curve of the ABCD^2^ score. **(E)** The combined ROC curve of miR-194-5p, SNHG1, and carotid artery stenosis degree.

The cutoff value for SNHG1 (*p* < 0.001; AUC = 0.836) was 0.810, with a sensitivity of 79.10%, specificity of 79.51%, negative predictive value (NPV) of 87.39%, and positive predictive value (PPV) of 67.95% (Figure 3A). The cutoff value for miR-194-5p (*p* < 0.001; AUC = 0.754) was 0.805, with a sensitivity of 64.18%, specificity of 74.59%, NPV of 79.13%, and PPV of 58.11% (Figure 3B). The cutoff value for carotid artery stenosis degree (*p* < 0.001; AUC = 0.884) was 41.91, with a sensitivity of 77.61%, specificity of 88.52%, NPV of 87.80%, and PPV of 78.79% (Figure 3C). The cutoff value for the ABCD^2^ score (*p* < 0.001; AUC = 0.875) was 4.182, with a sensitivity of 83.58%, specificity of 80.33%, NPV of 89.91%, and PPV of 70.00% (Figure 3D). However, the combined cutoff value for the three factors (*p* < 0.001; AUC = 0.953) was 0.350, with a sensitivity of 89.55%, specificity of 86.89%, NPV of 93.81%, and PPV of 86.89% (Figure 3E).

### Targeted binding of SNHG1/miR-194-5p

**Figure 4:**
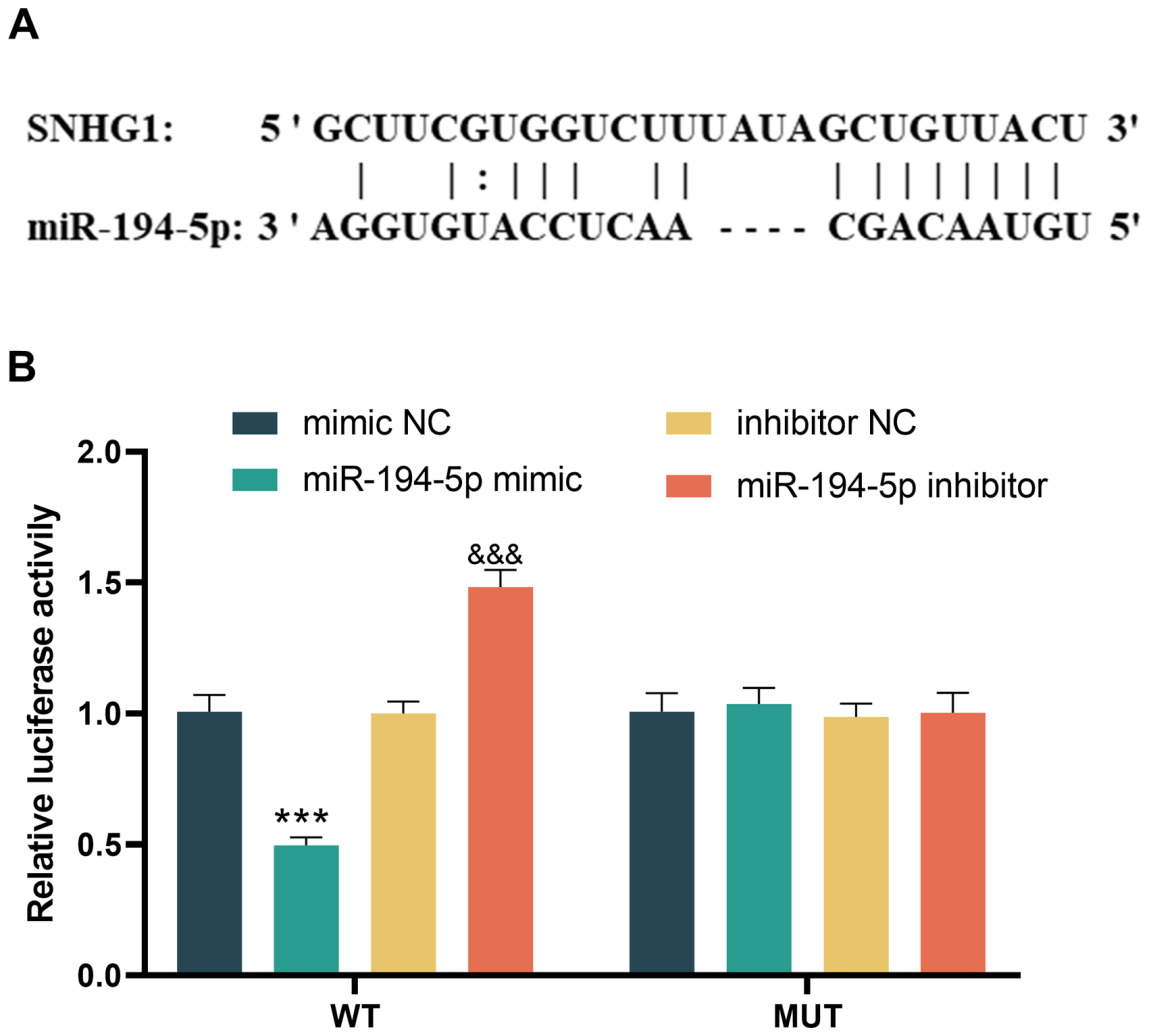
SNHG1 and miR-194-5p have targeted sites. **(A)** The bioinformatics database starBase identified the binding sequences of SNHG1 and miR-194-5p. **(B)** Dual-luciferase reporter gene assay was used to verify the targets between SNHG1 and miR-194-5p in SH-SY5Y cells (*** *p* < 0.001 compared to mimic NC; &&& *p* < 0.001 compared to inhibitor NC).

The presence of binding sites between SNHG1 and miR-194-5p was predicted using starBase (Figure 4A). The subsequent dual-luciferase reporter gene assay showed that overexpression of miR-194-5p inhibited the relative activity of luciferase in SH-SY5Y cells, while the reduction of miR-194-5p produced the opposite effect in the wild-type group (*p* < 0.01, Figure 4B). However, no significant change in luciferase activity was observed in the mutant group (all *p* > 0.05).

### Target prediction of miR-194-5p and construction of the PPI network

**Figure 5:**
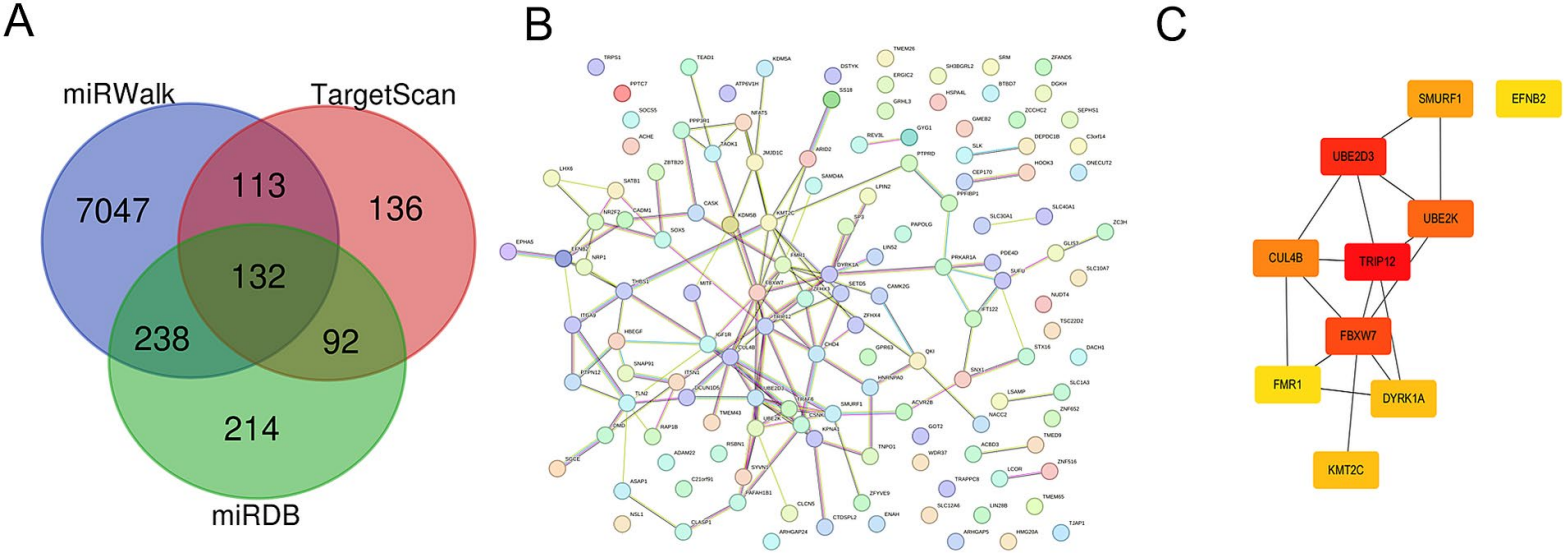
Target prediction and function analysis of miR-194-5p. **(A)** A total of 132 overlapping targets of miR-194-5p were identified in the TargetScan, miRWalk, and miRDB databases. **(B)** The PPI network hub genes. **(C)** The top 10 overlapping target genes.

To deeply explore the role of miR-194-5p in CI, we predicted the target genes of miR-194-5p using the TargetScan, miRWalk, and miRDB databases. As shown in Figures 5A, a total of 132 overlapping target genes were identified (Supplementary Table 1). Subsequently, these genes were imported into the STRING database to construct the protein–protein interaction (PPI) network (Figure 5B). The hub genes in the PPI network were identified using Cytoscape software based on the connectivity algorithm in the network and included TRIP12, UBE2D3, FBXW7, UBE2K, CUL4B, SMURF1, DYRK1A, KMT2C, EFNB2, and FMR1 (Figure 5C).

### Enrichment analysis of miR-194-5p target genes

**Figure 6:**
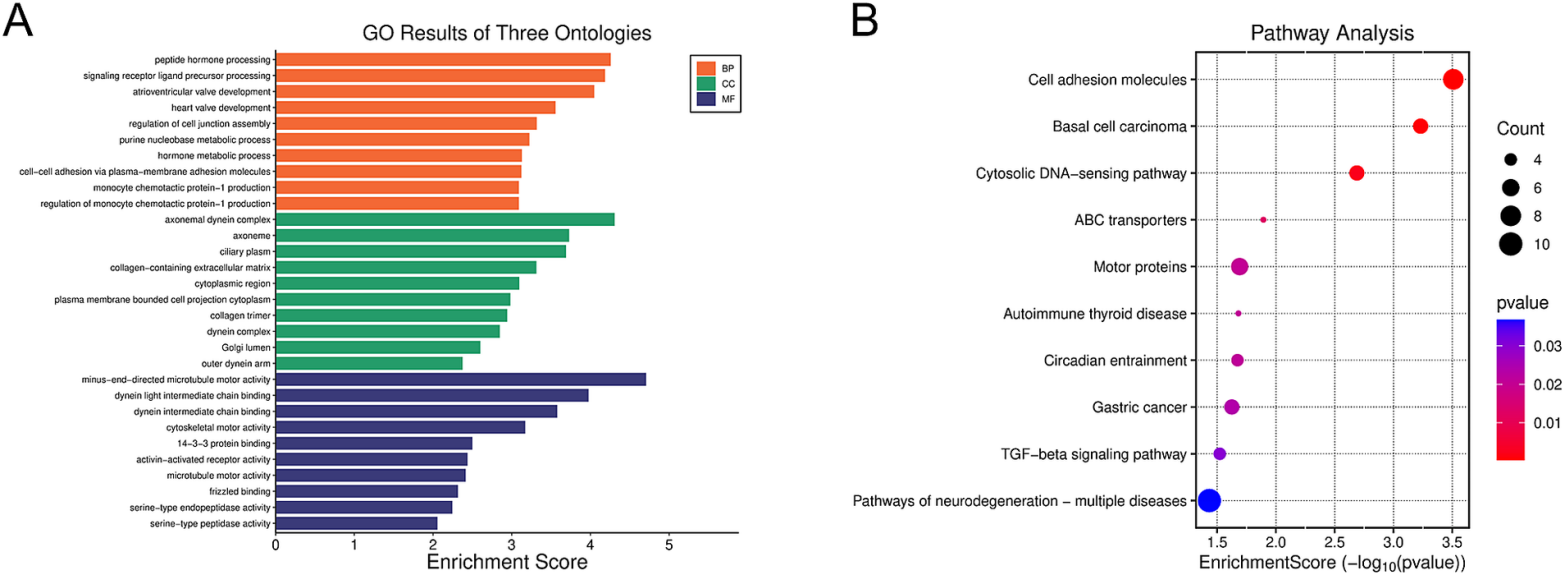
Enrichment analysis of miR-194-5p target genes using GO and the KEGG. **(A)** GO analysis. **(B)** KEGG pathway analysis.

Further GO and KEGG analyses were conducted on these overlapping target genes. As shown in Figure 6A, GO enrichment analysis revealed that biological processes (BPs) were primarily enriched in peptide hormone processing and signaling receptor ligand precursor processing. The molecular function (MF) process was primarily enriched in terminal-end-directed microtubule motor activity and dynein light intermediate chain binding. The cellular component (CC) process was primarily enriched in the axonemal dynein complex. KEGG pathway enrichment analysis indicated that these overlapping genes were significantly enriched in pathways related to multiple neurodegenerative diseases, cell adhesion molecules, and cytokine–cytokine receptor interactions (Figure 6B).

## Discussion

In this study, we constructed a combined prediction model for secondary CI based on circulating SNHG1/miR-194-5p levels and carotid ultrasound findings, which improved the accuracy and reliability of predicting CI risk in patients with transient cerebral ischemia. Additionally, we predicted the target genes of miR-194-5p and performed functional and related signaling pathway enrichment analyses, which can provide novel insights into the exploration of key genes involved in the development of CI.

SNHG1, a long non-coding RNA, may be involved in the occurrence and development of CI through multiple pathways (Yang et al., 2021). Studies have found that SNHG1 can adsorb miRNAs and relieve their inhibitory effects on its target genes, thereby regulating processes such as proliferation, invasion, and metastasis of tumor cells (Xu et al., 2018; Zhao et al., 2023). Similarly, in cerebrovascular diseases, SNHG1 may interact with miR-194-5p, influencing related signaling pathways and increasing the occurrence of CI. Studies have shown that miR-194-5p exerts neuroprotective effects in cerebral ischemia–reperfusion injury models by regulating a series of target genes related to neuronal apoptosis and inflammatory responses (Zhang et al., 2021). However, SNHG1 may reduce the expression level of miR-194-5p, weaken its regulatory effect on target genes, promote neuronal apoptosis, intensify inflammatory responses, and thereby increase the risk of CI. The results of this study showed that the relative serum expression level of SNHG1 in the CI group was significantly higher than that in the non-CI group, while the relative expression level of miR-194-5p was significantly lower. This indicates that circulating SNHG1/miR-194-5p levels are closely associated with CI occurrence. Furthermore, Pearson correlation analysis confirmed that the expression level of SNHG1 was negatively correlated with that of miR-194-5p.

In clinical practice, a single biomarker is often insufficient for the accurate diagnosis of complex diseases (Shi et al., 2021). The degree of carotid artery stenosis is one of the important risk factors for CI, and it can lead to insufficient cerebral blood supply, increase the risk of thrombosis, and thereby elevate the risk of CI (Wang et al., 2016; Jin, 2021; Reiff et al., 2022). Changes in serum SNHG1 and miR-194-5p levels may reflect the potential pathophysiological alterations in the body, interact with carotid artery stenosis degree, and jointly affect the risk of CI. The results of logistic regression analysis showed that the degree of carotid artery stenosis and serum SNHG1 levels were positively correlated with CI, while the serum miR-194-5p level was negatively correlated with CI. Meanwhile, the corresponding regression equation based on these three indicators was *p* = 1/[1 + e^-(−6.743 + 0.142×1 + 3.257×2-2.709×3)^]. Combined detection of multiple indicators can comprehensively reflect different aspects of a disease and improve diagnostic sensitivity and specificity (Song et al., 2025). In this study, serum miR-194-5p, serum SNHG1, and carotid artery stenosis degree all exhibited predictive value for CI occurrence. Moreover, when these three indicators were detected together, the AUC of the combined detection model was as high as 0.953, which was significantly superior to the predictive efficacy of any single indicator (all *p* < 0.05). Meanwhile, the predictive performance of this combined detection model was also better than that of the traditional ABCD^2^ score for CI prediction (AUC = 0.875), suggesting that the combined detection of these three factors has a more optimal clinical application value in CI prediction. This result provides a novel strategy for the early clinical diagnosis of CI.

In this study, we confirmed the targeted regulatory relationship between SNHG1 and miR-194-5p through starBase prediction and dual luciferase reporter gene assay. This finding provides significant insights into the regulatory network involving non-coding RNAs. We then attempted to analyze the potential molecular mechanisms by which miR-194-5p affects CI. In total, 132 overlapping target genes were predicted. In addition, a PPI network was constructed for the overlapping target genes. Cytoscape software was used to identify the top 10 hub genes, namely DYRK1A, SMURF1, UBE2K, EFNB2, TRIP12, FMR1, CUL4B, KMT2C, UBE2D3, and FBXW7. Among these genes, DYRK1A, UBE2K, FMR1, and FBXW7 have all been studied in the context of CI. In functional studies related to DYRK1A, it has been clearly demonstrated that it plays a role in repairing nervous system injuries. For instance, Demyanenko and Uzdensky (2021) confirmed that DYRK1A exerts neuroprotective effects in the penumbra of ischemic stroke by participating in various signal transduction pathways, regulating neurodegenerative-related proteins, promoting axon growth and guidance, and maintaining vesicle transport. He et al. (2023) experimentally demonstrated using a middle cerebral artery occlusion (MCAO) mouse model that miR-192-5p can inhibit the expression of the DYRK1A mRNA by targeting the 3’UTR region, thereby reducing neuronal apoptosis, lowering the levels of neuroinflammatory factors, and significantly alleviating brain injury in mice. We speculate that miR-194-5p may regulate the expression of DYRK1A by targeting it, thereby influencing the neuroprotective pathways mediated by DYRK1A (such as signal transduction and inhibition of neuronal apoptosis), and ultimately playing a role in the occurrence and development process of CI diseases. Further GO enrichment analysis indicated that these target genes were enriched in biological processes, molecular functions, and cellular component pathways. Biological processes (BP) were primarily enriched in peptide hormone processing and signaling receptor ligand precursor processing, both of which involve the precise regulation of intercellular signal communication. The MF process is primarily enriched in terminal-end-directed microtubule motor activity and dynein light intermediate chain binding. The cellular component (CC) process is primarily enriched in the axonemal dynein complex. Microtubules, the core cytoskeletal components of axons and dendrites in nerve cells, directly regulate the maintenance of nerve cell morphology through their motility activity and protein-binding function (Grewal et al., 2025). In the KEGG pathway enrichment analysis, they were significantly enriched in pathways related to multiple neurodegenerative diseases, cell adhesion molecules, and cytokine–cytokine receptor interactions. The enrichment analysis results further supported the hypothesis that “miR-194-5p may affect the occurrence and development of CI by regulating target genes and participating in the growth, development, apoptosis, and inflammatory response of nerve cells.”

This study provided certain insights into the predictive value of circulating SNHG1/ miR-194-5p and carotid ultrasound examination for the occurrence of CI in patients with transient cerebral ischemia; however, there are also some limitations.

In terms of sample size, the number of patients with transient cerebral ischemia included in this study was relatively limited, and no prospective study with a sample size efficacy design was conducted for the predictive efficacy of the biomarkers (SNHG1/miR-194-5p), which may have led to certain deviations in the research results. Although the AUC value of the joint prediction model was exceptionally high in this study, the relatively small dataset introduces a potential risk of overfitting, which may limit the model’s applicability in clinical practice and hinder the demonstration of its generalizability. The next step could be to expand patient recruitment through multi-center collaboration and to extend the follow-up period to improve the reliability of the results. Furthermore, the occurrence of CI is not only related to the regulation of ncRNAs and changes in vascular anatomy but also closely associated with the inflammatory state of the body and abnormal coagulation function. The absence of these indicators suggest that the current model does not fully capture the complete risk dimension of “molecular regulation – vascular structure – inflammatory response – coagulation function,” which may result in underestimating or overlooking some high-risk patients and limit the clinical comprehensiveness and predictive accuracy of the model. Subsequently, we aim to enhance our approach by supplementing the detection of serum inflammatory factors (CRP, IL-6) and coagulation function indicators (D-dimer, fibrinogen), incorporating these indicators into the predictive model for iterative optimization, and ultimately constructing a comprehensive predictive model covering multiple dimensions of risk, which enhances the early identification and clinical utility of cerebral infarction after transient ischemic attack.

In conclusion, we established a new regression equation for predicting CI based on the level of circulating SNHG1/miR-194-5p and carotid artery stenosis degree. SNHG1, miR-194-5p, and carotid artery stenosis degree can be used as diagnostic markers for cerebral infarction in patients with transient cerebral ischemia. The ROC curve AUC for the combined use of SNHG1, miR-194-5p, and carotid artery stenosis degree was higher. Furthermore, bioinformatics analysis revealed that the target genes of miR-194-5p were enriched in multiple disease pathways, especially those related to neurodegenerative diseases, providing a new direction for exploring the mechanism of CI.

## Data Availability

The original contributions presented in the study are included in the article/Supplementary material, further inquiries can be directed to the corresponding author.

## Footnotes

^1^ https://starbase.sysu.edu.cn/

^2^ http://www.bioinformatics.com.cn/
